# Evaluation of Daptomycin Use in Outpatients With Methicillin-Sensitive *Staphylococcus aureus* Bloodstream Infections

**DOI:** 10.1093/ofid/ofaf012

**Published:** 2025-01-17

**Authors:** Daisy Kener, Darrell Childress, Ian Andrus, Jared Olson, Brandon Webb

**Affiliations:** Department of Pharmacy, Intermountain Health Utah–St George Regional Hospital, St George, Utah, USA; Department of Pharmacy, Intermountain Health Utah–St George Regional Hospital, St George, Utah, USA; Department of Pharmacy, Intermountain Health Utah–St George Regional Hospital, St George, Utah, USA; Department of Pharmacy, Intermountain Health Utah–Primary Children's Hospital, Salt Lake City, Utah, USA; Division of Pediatric Infectious Diseases, University of Utah, Salt Lake City, Utah, USA; Division of Infectious Diseases, Intermountain Medical Center, Salt Lake City, Utah, USA

**Keywords:** bacteremia, blood stream infection, BSI, daptomycin, methicillin-sensitive *Staphylococcus aureus*, MSSA

## Abstract

Multiple observational studies in methicillin-resistant *Staphylococcus aureus* and enterococcal infections have suggested that higher doses of daptomycin may be associated with better clinical outcomes. However, optimal daptomycin dosing in methicillin-sensitive *S aureus* bloodstream infections remains unclear. In this multicentered, retrospective, observational cohort study, we compared standard dose daptomycin (<8 mg/kg) vs high dose (≥8 mg/kg) for methicillin-sensitive *S aureus* bloodstream infections. In a propensity-weighted model, the composite outcome of treatment failure within 90 days was lower in the high-dose group relative to the standard dose group (odds ratio, 0.496; 95% CI, .306–.804). We did not detect any significant difference in safety outcomes.

*Staphylococcus aureus* is the leading cause of bacteremia and is associated with higher rates of morbidity, mortality, and complicated infections [1–3]. Antistaphylococcal β-lactams (ASBLs) such as nafcillin, oxacillin, and cefazolin (first-generation cephalosporin) are the preferred treatment for methicillin-sensitive *S aureus* (MSSA) bacteremia. However, these agents require multiple administrations per day and are not a viable option in some outpatient parenteral antibiotic therapy (OPAT) settings. Daptomycin is a cyclic lipopeptide antibiotic that is bactericidal in a concentration-dependent manner [4]. Daptomycin has several advantages, including once-daily administration and a similarly favorable safety profile as ASBLs, and unlike cefazolin, it may not be prone to in vitro inoculum effect against some MSSA strains [5, 6]. Daptomycin is Food and Drug Administration approved for the treatment of *S aureus* bloodstream infections (BSIs) at a dose of 6-mg/kg intravenous infusion every 24 hours [4].

Several studies have suggested that higher doses of daptomycin are safe: ≥8 to 10 mg/kg for methicillin-resistant *S aureus* (MRSA) and ≥8 to 12 mg/kg for vancomycin-resistant *Enterococcus* spp. May be associated with better clinical outcomes, and they have been shown to be appropriate [7–12]. Fowler et al performed an open-label study that compared daptomycin with the standard therapy for bacteremia and endocarditis staphylococcus infections and concluded that daptomycin at 6 mg/kg once daily was noninferior to the standard therapy in bacteremia and right-sided endocarditis caused by MRSA or MSSA [13]. A single retrospective study also concluded that daptomycin (median dose, 7 mg/kg) may be comparable to the ASBLs in the treatment of MSSA BSIs, as there were no differences in the clinical outcomes [14]. Chastain et al compared the efficacy of antimicrobial therapies used in the management of persistent MSSA BSIs and suggested that daptomycin might be an alternative in patients with persistent bacteremia [15]. Similarly, a study evaluated the efficacy of high doses of daptomycin (≥8 mg/kg) vs lower doses (4 to 7 mg/kg) and found better treatment success with the higher doses [16].

However, optimal daptomycin dosing in MSSA BSI is not as well established. To address this important knowledge gap, we conducted a multicenter, retrospective, observational cohort study to evaluate whether composite treatment failure was improved in patients with MSSA BSI who received high-dose daptomycin (≥8 mg/kg) vs those who received standard dosing (<8 mg/kg).

## METHODS

**Figure 1.**
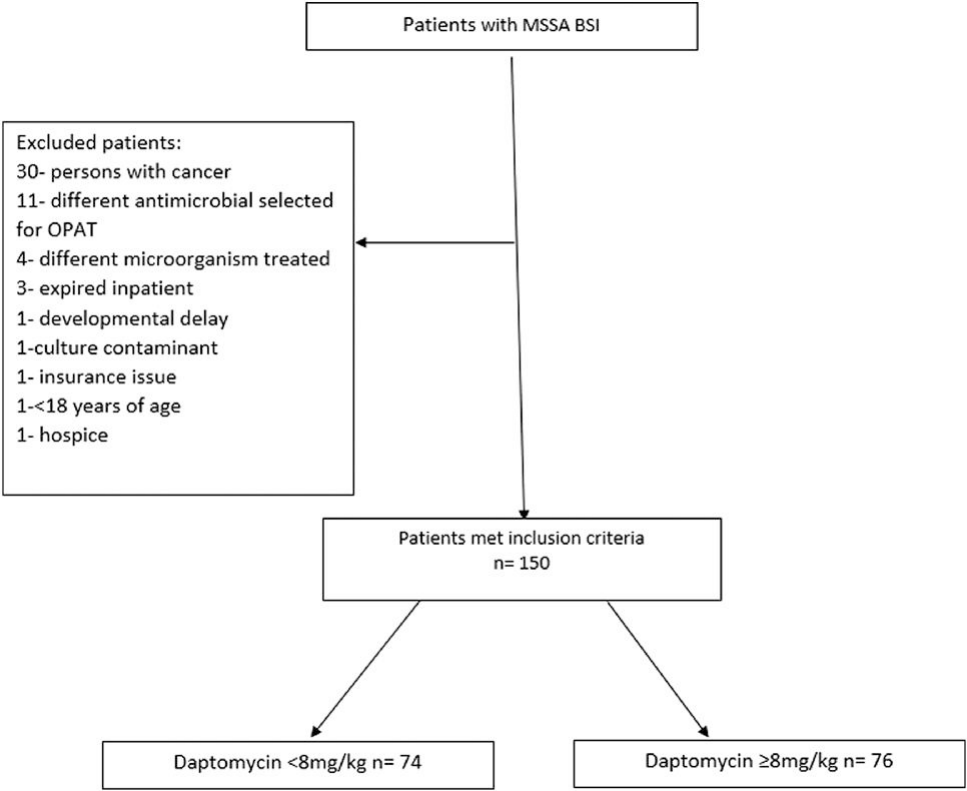
Patients identified to fit the inclusion criteria. BSI, bloodstream infection; HD, high dose; MSSA, methicillin-sensitive *Staphylococcus aureus*; OPAT, outpatient parenteral antibiotic therapy; SD, standard dose.

This institutional review board–approved multicenter, retrospective, observational cohort study was conducted in the Intermountain Health integrated health system. We included all adult patients admitted with MSSA BSI to 1 of 22 Intermountain hospitals between 1 January 2010 and 31 October 2023 who had an OPAT discharge with daptomycin. A combination of electronic and manual chart review was then performed with 2 types of electronic health record software: iCentra (Cerner) and Help2. Electronic data such as admission, discharge, dosing, and laboratory values were pulled by 1 of the investigators. A manual chart review was conducted to include data that could not be collected electronically and to confirm diagnosis, discharge, and readmission information. Three independent reviewers followed the data collection protocol with any ambiguity being addressed with the primary investigator. Patients were excluded if they were being actively treated for malignancy, died in the hospital, or were discharged with hospice, due to the potential to skew outcomes (Figure 1). Protected health populations were also excluded. The study population was divided into 2 study groups based on their dose of daptomycin at discharge: standard dose (SD; <8 mg/kg) and high dose (HD; ≥8 mg/kg) according to adjusted body weight (ABW). ABW was calculated with following equation: ideal body weight + 0.4 × (ABW − ideal body weight).

The primary outcome was composite treatment failure within 90 days of discharge, defined as any of the following: infection-related readmission, MSSA BSI recurrence, change of antimicrobial agents, and/or mortality [6]. Secondary outcomes included highest observed creatine kinase (CK) elevation, incidence of diagnosed eosinophilic pneumonia, and comparison of ABW vs total body weight in persons with obesity (body mass index ≥30 kg/m^2^).

### Statistical Analysis

To mitigate indication bias, we developed a propensity model by fitting a logistic regression model with HD vs SD as the dependent variable and with the following adjuster covariates: age, weight, body mass index, sex, race, Charlson Comorbidity Index, Pitt score, follow-up blood cultures with positive results beyond 48 hours, serum creatinine, statin use, primary source of infection, and intensive care unit admission during hospitalization. The primary source of infection was determined by the diagnosis of endocarditis, and for those patients without endocarditis, the primary source of infection was used as the diagnosis. Those without any endocarditis or other known infection were labeled as infections of unknown source. Additionally, complicated bacteremia was defined as patients with positive blood culture results and any of the following: endocarditis, implanted prostheses, positive follow-up blood cultures obtained 48 to 96 hours after the initial set, continued fevers past 72hours, and evidence of metastatic sites of infection [7]. Uncomplicated bacteremia was defined as none of the aforementioned. The subcomponents of complicated bacteremia were also integrated into our propensity model. Source control was defined as surgical intervention and/or negative follow-up blood culture results. Because hospital site was strongly collinear with daptomycin dose selection due to variation among hospital-based infectious disease practices, inclusion of hospital in the propensity model led to nonconvergence and obscured the effect of other variables. We therefore conducted a prespecified sensitivity analysis by fitting a separate propensity model including hospital sites as the only adjuster variable to determine if unmeasured confounders due to other differences in practice that vary by site may be influencing the outcome.

The primary and secondary analyses were then conducted by fitting a logistic regression model with the propensity score to perform inverse probability of treatment weighting (IPTW). Weights were truncated at their 2.5th and 97.5th percentiles to prevent outliers from influencing effect estimates. Standardized mean differences in confounder variables between groups were plotted before and after IPTW. Statistical analysis was performed with R version 4.2.3 (R Project for Statistical Computing).

## RESULTS

Between January 2010 and October 2023, 150 patients met inclusion criteria, among whom the composite treatment failure at 90 days occurred in 66 (44%). Individual components of the primary outcome were as follows: readmission, 62 patients (41%); mortality, 6 (4%); recurrence of bacteremia, 8 (5%); and antibiotic change, 9 (6%). Adverse events were rare: CK abnormalities occurred in 5 patients (3%), and a diagnosed incidence of eosinophilic pneumonia occurred in 1 patient (1%) who received HD daptomycin. Of the 150 patients, 74 (49%) received SD and 76 (51%) received HD. Overall, 3 patients in the HD group had an SD dosing while hospitalized; all other daptomycin doses were the same at discharge. Groups were similar in terms of age, gender, weight, and complications of infection (Table 1). Patients in the HD group had more comorbidities but lower frequency of intensive care unit stays. After IPTW, standardized mean differences were minimized to <0.1 in all variables of interest.

**Table 1.** Baseline Characteristics and Primary and Secondary Outcomes

	Unadjusted, No. (%) or Median (IQR)				Propensity-Weighted Cohort, % or Median (IQR)			
	SD (n = 74)	HD (n = 76)	*P* Value	SMD	SD (n = 146.3)	HD (n = 146.4)	*P* Value	SMD
Site			<.001				<.001	
Telehealth	11 (14.9)	14 (18.4)		0.035	12.3	20.9		0.086
IMC	5 (6.8)	17 (22.4)		0.156	6.5	23.1		0.166
McKay-Dee Hospital	19 (25.7)	25 (32.9)		0.072	28.2	26.3		−0.019
St George Regional Hospital	37 (50.0)	6 (7.9)		−0.421	51.3	10.0		−0.413
Utah Valley Hospital	2 (2.7)	14 (18.4)		0.157	1.6	19.7		0.181
Female	22 (29.7)	29 (38.2)	.359	0.179	33	34.9	.835	0.019
Race			.496				.957	
White	66 (89.2)	66 (86.8)		−0.024	90.4	89.4		−0.01
Unknown	4 (5.4)	6 (7.9)		0.025	5.0	6.1		0.011
Age, y	61.5 (43.00–70.75)	58.5 (43.00–71.25)	.826	0.033	60.36 (43.0–71.56)	59.0 (45.0–71.0)	.915	0.011
Pitt score	1 (0.00–1.00)	1 (1–2.25)	.077	0.357	1 (1–2)	1 (0–2)	.918	0.035
CCI	3 (1–6)	4 (2–6)	.217	0.178	4 (1–6.91)	4 (2–6)	.820	0.032
Weight, kg	83.8 (72.00–97.43)	90.75 (75.9–106.82)	.06	0.285	86 (72.98–98.49)	90.25 (74.69–104.9)	.453	0.089
Adjusted body weight, kg	75.98 (66.84–83.35)	79.50 (65.69–86.51)	.243	0.148	75.97 (67.8–84.17)	80.23 (65.66–85.69)	.482	0.066
Serum creatinine	1.03 (0.78–1.48)	0.93 (0.80–1.24)	.180	0.391	0.95 (0.76–1.36)	0.97 (0.83–1.39)	.581	0.007
Daptomycin								
Dose, mg	500 (405–590)	700 (600–800)	<.001	1.635	500 (400–600)	700 (600–800)	<.001	1.494
Dose, mg/kg, adjusted body weight	6.54 (6.06–7.20)	9.02 (8.33–9.88)	<.001	2.913	6.52 (6.03–7.22)	8.83 (8.26–9.73)	<.001	2.752
Positive follow-up blood culture ≥48 h	9 (12.2)	7 (9.2)	.748	−0.03	9.7	9.9	.959	0.002
Infectious disease consult	68 (91.9)	71 (93.4)	.963	0.015	92.1	93.6	.733	0.015
Documented negative blood culture	71 (95.9)	75 (98.7)	.593	0.03	97.6	98.7	.567	0.011
Source of infection			.844				>.99	
Central nervous system	8 (10.8)	8 (10.5)		−0.003	12.7	12.6		−0.001
SSTI/DFI	21 (28.4)	18 (23.7)		−0.047	27.7	25.9		−0.018
Gastrointestinal/genitourinary	5 (6.8)	4 (5.3)		−0.015	4.6	4.3		−0.003
Infective endocarditis	3 (4.1)	6 (7.9)		0.038	5.0	5.9		0.009
IOUS/IVDU	14 (18.9)	11 (14.5)		0.044	16.4	17.0		0.006
Osteo/joint	10 (13.5)	15 (19.7)		0.062	15.5	15.4		−0.001
Line	13 (17.6)	14 (18.4)		0.008	18.2	18.9		0.007
Intensive care unit	10 (13.5)	6 (7.9)	.395	−0.056	13.7	12.3	.844	−0.014
Complicated infection	56 (75.7)	57 (75)	>.99	−0.007	73.1	76.7	.655	0.036
No. of source control interventions	0 (0–1)	1 (0–1)	.088	0.276	0 (0–1)	1 (0–1)	.357	0.157
Statin	18 (24.3)	22 (28.9)	.649	0.046	25.6	25.1	.951	−0.005
Length of stay, d	6 (4–9)	6 (4.75–8)	.654	0.093	6 (4–9)	6 (4–8.27)	.762	0.091

Abbreviations: CCI, Charlson comorbidity index; DFI, diabetic foot infection; HD, high dose; IMC, Intermountain Medical Center; IOUS, unknown source of infection; IVDU, intravenous drug use; SD, standard dose; SMD, standardized mean difference; SSTI, skin soft tissue infection.

Unadjusted composite treatment failure at 90 days occurred in 27 (36%) and 39 (53%) patients in the HD and SD groups, respectively. In the primary analysis (Table 2), composite treatment failure at 90 days was lower in the HD group relative to the SD group (odds ratio [OR], 0.496; 95% CI, .306–.804). The individual components of the composite outcome within 90 days were as follows: readmission (OR, 0.516; 95% CI, .317–.839), mortality (OR, 0.356; 95% CI, .0713–1.780), recurrence of bacteremia (OR, 0.382; 95% CI, .130–1.124), and antibiotic change (OR, 0.456; 95% CI, .166–1.252). Results were robust to the prespecified sensitivity analysis (OR, 0.61; 95% CI, .39–.97).

**Table 2.** IPTW Adjusted Primary and Secondary Outcomes

	Odds Ratio (95% CI)
Primary end point	0.496 (.306–.804)
90 d	
Readmission	0.516 (.317–.839)
Death	0.356 (.0713–1.780)
Recurrence	0.382 (.130–1.124)
Antimicrobial change	0.456 (.166–1.252)
CK >1000 U/L	0.920 (.227–3.724)

Abbreviations: CK, creatine kinase; IPTW, inverse probability of treatment weighting.

There was no difference between the HD and SD groups in regard to safety outcomes, including CK ≥1000 U/L (3.9% vs 2.7%, respectively; OR, 0.96; 95% CI, .24–3.86) and a single diagnosed incidence of eosinophilic pneumonia (1.3%) in the HD group and no cases in the SD group.

## DISCUSSION

Daptomycin offers an advantage as a once-daily 2- to 5-minute infusion in patients with MSSA BSI OPAT as compared with ASBLs [4]. Several studies have suggested HD daptomycin as compared with SD due to higher treatment success [13, 15–17]. The results from this study indicate that there was a decrease in treatment failure with HD daptomycin when compared with SD. Overall, given the mechanism of action of daptomycin, the *mecA* gene is irrelevant, and similar high daptomycin dosing strategies for MRSA infections should be applicable to MSSA infections.

Readmission was a major driver in the primary composite outcome with lower readmission in the HD group. Although the primary source of infection was not significant, it appears that higher doses of daptomycin were used in severe infections (joint, infective endocarditis, and unknown source of infection). In terms of safety, there was no difference between the groups; however, we had a small sample size.

This study is not without limitations, given the retrospective nature and cohort study design, as well as the fact that not all confounding factors could be fully mitigated. There was potential incomplete data due to transfer between 2 electronic health records. Our assumption of source control was either surgical intervention and negative follow-up blood culture results or those who did not have surgical intervention and negative follow-up blood culture results. Overall, the SD group had documented clearance of BSI in 95.9% of patients as compared with the HD group with 98.4% (*P* = .6). Additionally, the primary treatment received before discharge with daptomycin OPAT could have affected the primary outcome as well. This study had a small sample size and a less diverse patient population. We did not evaluate cost-effectiveness, which is another important consideration.

Future studies are warranted to evaluate CK, incidences of diagnosed eosinophilic pneumonia in a larger population, and optimal dosing management in persons with obesity (body mass index >40 kg/m^2^).

## CONCLUSION

There was a statistically significant difference in the composite outcome: 90-day treatment failure between the HD and SD groups. The HD daptomycin group demonstrated lower treatment failure, with less readmission being the major driving factor. The secondary safety outcomes were similar between the groups. Overall, the results from this study suggest that HD daptomycin may be preferred in patients discharged with OPAT for MSSA BSIs.

## Notes

***Acknowledgments.*** We acknowledge Amy S. Childress, MA, for technical formatting of the manuscript.

***Financial support.*** All authors state that this work was not directly supported by any funding agencies.

***Patient consent statement.*** The design of the work has been approved by Intermountain Health institutional review board ethical committees, or it conforms to standards currently applied in the country of origin and includes the name of the authorizing body stated in the brief report.
